# Enzymatic Acrolein Production System and Its Impact on Human Cells

**DOI:** 10.1021/acs.chemrestox.4c00119

**Published:** 2024-07-09

**Authors:** Katherine A. Hurley, Jacob Folz, Jasmin Zgraggen, Tania N. Cruz, Sabine Diedrich, Shana J. Sturla

**Affiliations:** Department of Health Sciences and Technology, ETH Zürich, Zurich 8092, Switzerland

## Abstract

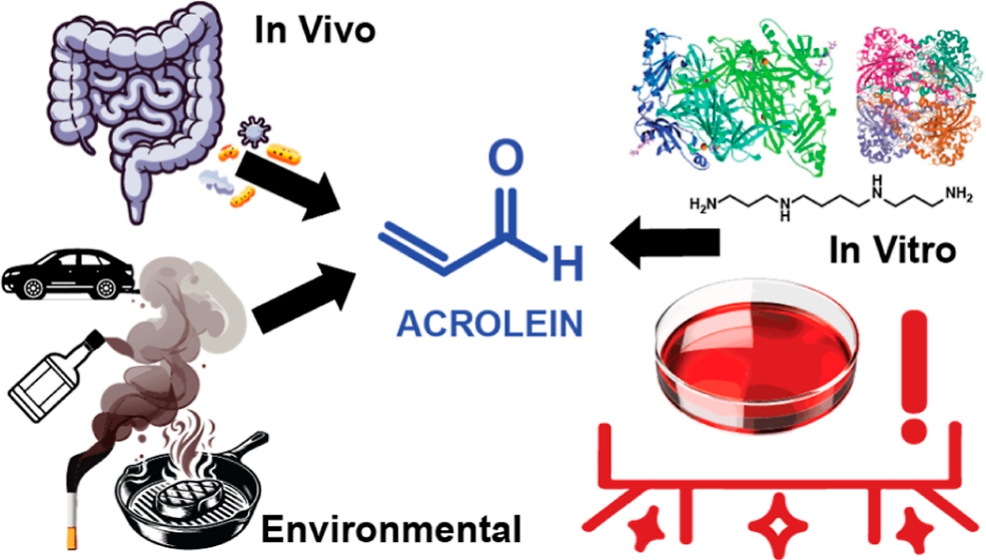

Acrolein is an environmental toxicant and is also generated by microbial metabolism in the intestinal tract. Aqueous acrolein rapidly dissipates from standard human cell culture media with nondetectable levels after 8 h, hindering cell-based studies to understand its biological impacts. Thus, we developed an extracellular acrolein biosynthesis system to continuously produce acrolein compatible with human cell culture conditions. The approach uses spermine as a precursor, amine oxidase found in fetal calf serum, and catalase to remove the hydrogen peroxide byproduct. We confirmed amine oxidase activity of calf serum using a colorimetric assay and further tested the requirement for catalase in the system to mitigate hydrogen peroxide-induced cytotoxicity. We calibrated responses of human colon cells to this enzymatic acrolein production system by comparing transcriptional responses, DNA adduct formation and cytotoxicity responses to either this system or pure acrolein exposures in a human colon cell line. Several genes related to oxidative stress including HMOX1, and the colorectal cancer-related gene SEMA4A were upregulated similarly between the enzymatic acrolein production system or pure acrolein. The acrolein-DNA adduct γ–OH-Acr-dG increased in a dose-dependent manner with spermine in the enzymatic acrolein production system, producing a maximum of 1065 adducts per 10^8^ nucleosides when 400 μM spermine was used. This biosynthetic production method provides a relevant model for controlled acrolein exposure in cultured human cells and overcomes current limitations due to its physical properties and limited availability.

## Introduction

Gut microbes produce thousands of unique metabolites that can impact host health^1^ including microbially generated acrolein.^2,3^ This highly reactive chemical is also present in cigarette smoke, automobile exhaust, fried foods, and alcoholic beverages.^4^ Acrolein is classified by the International Agency for Research on Cancer as a probable human carcinogen (Group 2A), and associated with lung cancer, renal diseases, cerebral stroke, colon cancer, and Alzheimer’s disease.^4,5^ Oral acrolein exposure studies have not been carried out in humans, however in mice, oral acrolein exposure causes intestinal epithelial barrier damage, translocation of bacterial endotoxin-lipopolysaccharides into the bloodstream, endoplasmic reticulum stress-mediated apoptosis of epithelial cells and redistribution of tight-junction proteins.^6^ Adverse effects due to acrolein are thought to occur via chemical modification of nucleophilic biomolecules, including proteins and DNA, and promotion of oxidative stress,^7−10^ yet there are significant gaps concerning key relevant molecular and cellular processes in intestinal cells directly exposed to acrolein.^4,6,11,12^

**Figure 1:**
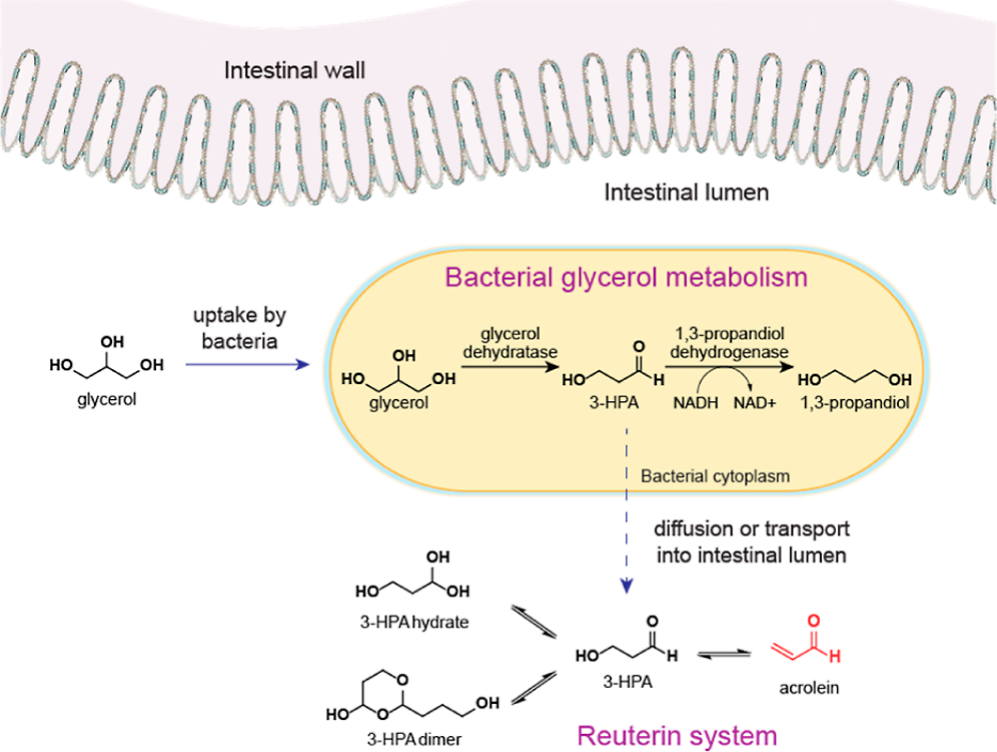
Bacterial glycerol metabolism occurs intracellularly and the formation of acrolein as a component of the reuterin system occurs in the lumen of the intestines.

In the context of the gut microbiome, acrolein is generated extracellularly in the gut after glycerol is converted by glycerol/diol dehydratase to 3-hydroxypropionic acid (3-HPA), which is excreted by microbes and degrades spontaneously to acrolein (Figure 1).^11−13^ 3-HPA is part of the reuterin system, a chemical equilibrium with the corresponding hydrate (1,1,3-propanetriol), its dimer (2-(2-hydroxyethyl)-4-hydroxy-1,3-dioxane), and acrolein (prop-2-enal) (Figure 1).^12^ Reuterin exhibits broad-spectrum antimicrobial activity, which requires acrolein, presumably due to the capacity of acrolein to react with nucleophilic biomolecules.^12^ The presence of GDH-active bacteria are common among gut microbiota, and linked to acrolein release,^14^ which leads to formation of acrolein-DNA, protein, and biomolecule adducts.^4^ Acrolein concentration in the intestine have been predicted to be from 58 μM to 7.8 mM acrolein depending on microbiota structure, with >70% of the acrolein expected to be in a bound state.^14^ Therefore, microbial acrolein in the human intestine may impact host cell function.

Acrolein is a reactive chemical that reacts with DNA, protein, and glutathione (GSH) to form acrolein adducts.^15−18^ This reactivity explains the detrimental effect that acrolein has on cells. Acrolein-DNA adducts are associated with the occurrence of lung, bladder, and colorectal cancer.^19−21^ The most prevalent acrolein-DNA adducts are formed by conjugate addition of guanosine, resulting in alpha-hydroxy-1, N_2_-propano-2′-deoxyguaninosine (α–OH-Acr-dG) and gamma-hydroxy-1, N_2_-propano-2′-deoxyguaninosine (γ–OH-Acr-dG) regioisomers.^8,22^ These adducts promote primarily G to T mutations,^19,23^ and adduct patterns in the tumor suppressor gene p53 correlate with p53 G to T mutations in lung cancer patients.^19^ Acrolein-protein adducts are proposed to contribute to many pathological impacts including cardiovascular and neurodegenerative diseases.^24^ For example acrolein disrupts cholesterol homeostasis and lipid metabolism by binding essential lysine residues within apoE to disrupt the structural and functional integrity of the protein.^25^ Acrolein can also disrupt transcription factors, such as the NF-κB, to inhibit protein–DNA interaction.^26^ Amino acids susceptible to acrolein binding are cysteine, histidine, and lysine that provide a broad potential to disrupt cellular functions.^4^ The antioxidant tripeptide GSH binds acrolein and the acrolein-GSH adducts (3-hydroxypropylmercapturic acid) are degraded as a mechanism to remove acrolein from the cell. Acrolein has been shown to reduce intracellular GSH levels, decrease antioxidant capacity, and decrease expression of antioxidant regulating enzymes in lung cells.^27^ Disruption of the oxidant/antioxidant levels in cells is characteristic of chronic lung disease.^28^ Understanding acrolein reactivity toward biomolecules, impacts on intestinal cells and contributions to disease etiology requires further research with exposure protocols suited to working with cell lines and other in vitro models.

To address gaps related to molecular and cellular effects of intestinal acrolein exposure, we established and characterized a cell-culture-compatible enzymatic acrolein production system for the continuous exposure of cultured human cells to acrolein. We employed amine oxidase enzymes present in standard cell culture media to convert exogenous spermine to acrolein and added a catalase enzyme to dissipate the toxic byproduct hydrogen peroxide. Individual components of this system were optimized for acrolein production and lack of cytotoxicity. The optimized system was then used to characterize cellular responses of a human colon cell line to acrolein, including cytotoxicity, gene transcription, and DNA adduct formation. Results from enzymatically produced acrolein were calibrated with the same end points from pure acrolein exposure to determine acrolein levels supplied by the enzymatic acrolein production system.

## Material and Methods

### Summary

Specific chemicals, procedures, and conditions for all experiments are described in the Supporting Information file. Briefly, **cell line** of human colorectal adenocarcinoma cells (SW480) were cultured as a monolayer in 10 cm dishes and transferred to 96 well plates. **Cell viability** was determined by addition of WST-1 cell proliferation reagent followed by absorbance measurement at 440 nm and normalized to media only treatment conditions. **Amine oxidase** levels were measured by hydrogen peroxide production adapting the diamine oxidase activity kit (Sigma-Aldrich). **Glutathione levels** were measured using the GSH/GSSG-Glo Assay kit (Promega). **Acrolein concentration** was measured by derivatization with 0.5 mM 4,5-dimethoxy-1,2-phenylenediamine hydrochloride (DDB) to form DDB-Acrolein (DDB-Acr) adducts that were measured by LC–MS/MS.^29^**DNA adducts** were quantified by hydrolysis of DNA followed by solid phase extraction and LC–MS/MS analysis. Total nucleosides were estimated based on quantified dG by HPLC for each sample. **Transcription levels** were measured by mRNA isolation with mini-RNeasy RNA isolation kits (Qiagen), retrotranscription using the High-Capacity cDNA Reverse Transcription Kit (ThermoFisher Scientific), qPCR performed using primers listed in supplementary methods, and transcript levels normalized to GAPDH.

### Enzymatic Acrolein Production System Method

SW480 cells were seeded in 10 cm plates in RPMI media with fetal calf serum (FCS) and incubated until a density of 80–90% confluency was reached (∼72 h). Spermine was dissolved in RPMI without FCS media, catalase was dissolved in 50 mM potassium phosphate buffer, and Hyclone FCS was defrosted and warmed to 37 °C. After old media was aspirated from culture dishes, cells were washed with 1xPBS, and the appropriate volume of new RPMI without FCS media was added to all dishes depending on treatment. Spermine stock solution and catalase were added dropwise to growth dishes, and the enzyme reaction was initiated by adding Hyclone FCS. The dishes were mixed by moving them in a Figure 8 pattern 5 times in both directions and incubated at 37 °C for 6 h. After exposure, cells were either tested for viability or scraped and stored at −80 °C until DNA or RNA extraction.

## Results and Discussion

### Development of a Cell-culture-compatible System to Produce Acrolein

**Figure 2:**
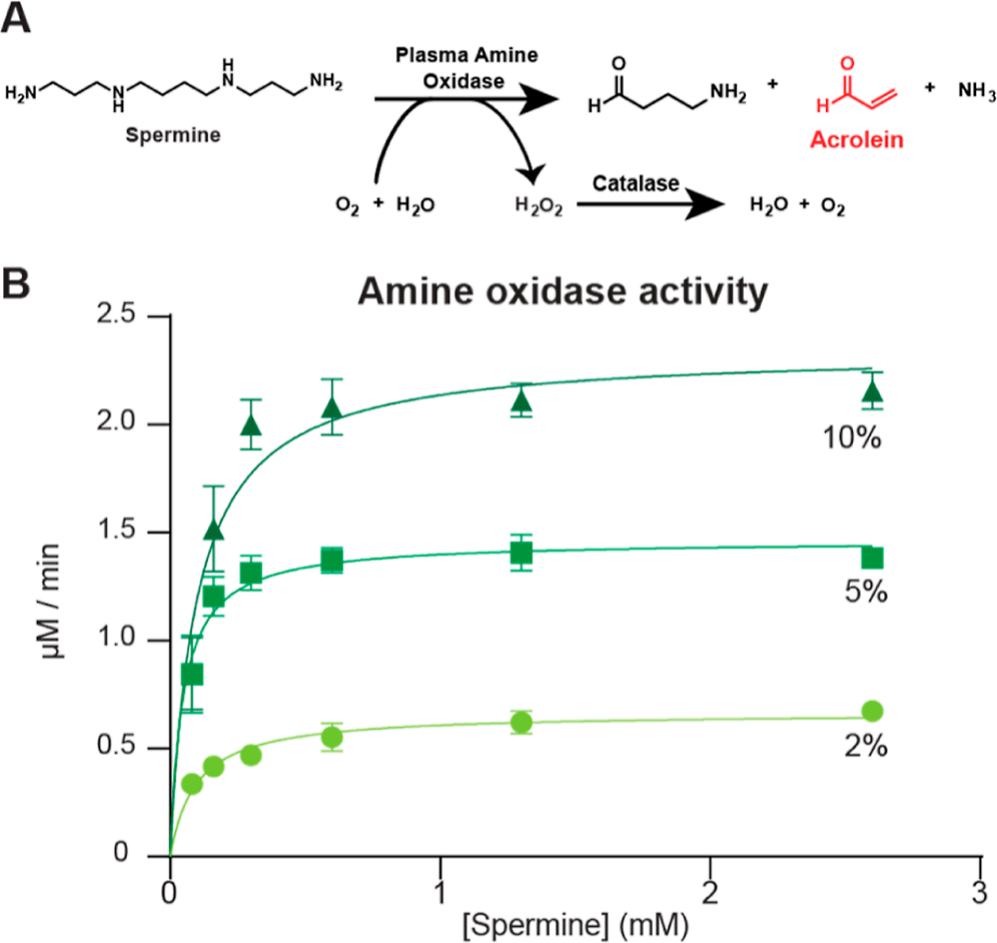
(A) Diagram of the enzymatic acrolein production system that produces acrolein and simultaneously degrades hydrogen peroxide. (B) Michaelis–Menten velocity curves of 3 different concentrations (2, 5 and 10%) of FCS with spermine in phosphate buffer monitored by colorimetric signal produced by hydrogen peroxide oxidizing *o*-dianisidine. The colorimetric signal was linear for less than an hour before saturation for 5 and 10% FCS at all spermine concentrations. For the 2% FCS condition, the signal was linear for the first 2 h before saturation for all the spermine concentrations.

In order to develop a cell-based system to maintain continuous concentrations of acrolein over time as occurs in the human intestinal tract, we decided to exploit enzymes to build an in vitro platform to model acrolein exposure. Acrolein is produced through three mammalian metabolism pathways: (1) myeloperoxidase excreted by human neutrophils convert hydroxy-amino acids into acrolein,^30^ (2) lipid peroxidation produces acrolein through a radical cascade reaction,^31^ and (3) catabolism of polyamines to acrolein by amine oxidases.^30,32,33^ We implemented the third pathway as an enzymatic acrolein production system involving simple reactant materials and amine oxidases present in the common cell growth media component calf serum. However, in the presence of FCS, spermine-associated cytotoxicity in cell culture experiments has been linked to the associated hydrogen peroxide production.^34−36^ Thus, we designed an enzymatic acrolein production system based on spermine transformation to acrolein by amine oxidase from calf serum, with removal of the coproduced hydrogen peroxide by catalase (Figure 2A).

Serum amine oxidase is a copper-containing enzyme that uses a topaquinone cofactor in a ping-pong mechanism to catalyze the oxidative deamination of polyamines to an aldehyde, ammonia, and hydrogen peroxide.^37−39^ More specifically in the case of spermine and spermidine, after oxidative deamination, the resulting aldehyde, 3-aminopropanal, spontaneously degrades to acrolein via β-elimination.^34,35,40,41^ Three-aminopropanal is a neurotoxin,^42^ however its spontaneous conversion to acrolein is expected to be the primary driver of cytotoxicity in this system.^35^ To evaluate the activity of amine oxidase in FCS, we measure the associated hydrogen peroxide production by coupled peroxidase-mediated oxidation of *o*-dianisidine, by colorimetric detection. For Hyclone FCS solutions of 2, 5 or 10%, which we found in preliminary tests to have high activity among different commercial sources, *V*_max_ values were 0.666, 1.46, and 2.34 μmol/min respectively (Figure 2B). These results suggested adjusting FCS concentrations as a strategy for controlled acrolein production in media. FCS concentrations of 2 and 5% were chosen for further development based on relevant acrolein concentrations relative to cell viability studies with pure acrolein.

### Cell Viability and Redox Capacity after Pure Acrolein Exposure

**Figure 3:**
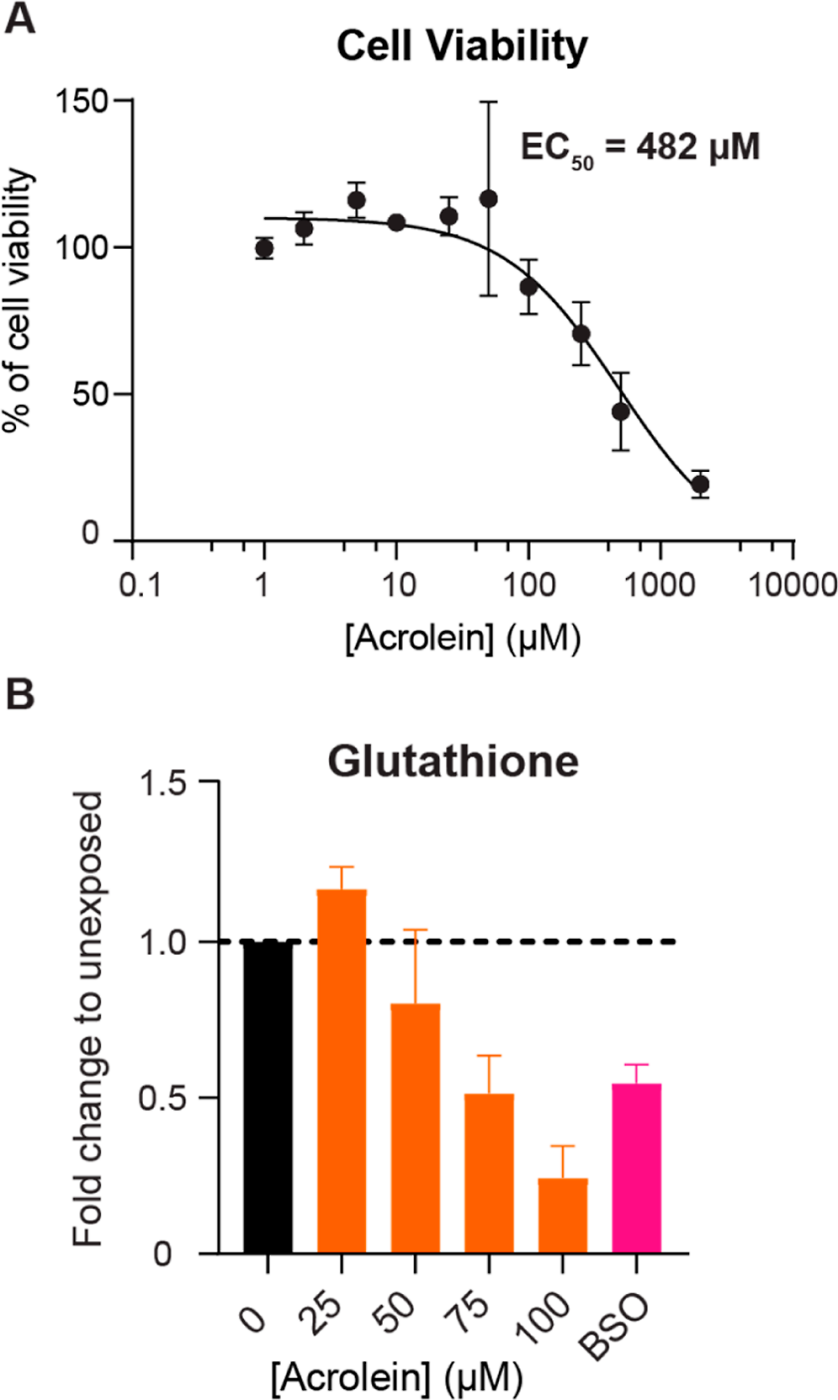
(A) Cell viability of SW480 cells after pure acrolein exposure. SW480 cells were exposed to acrolein between 0 and 100 μM in RPMI media without FCS for 6 h at 37 °C. (B) Fold change of glutathione levels in SW480 cells after pure acrolein exposure. SW480 cells were exposed to acrolein at concentrations between 0 and 100 μM in RPMI media without FCS for 6 h at 37 °C. dl-buthionine-(S,R)-sulfoximine (BSO) (500 μM), a glutathione synthase inhibitor, was used as a positive control. Fold change was calculated to unexposed SW480 cells in RPMI media without FCS and all conditions were normalized to cell count.

To calibrate the responses of a human colon cell line to defined concentrations of acrolein, we evaluated the cell viability of SW480 cells after exposure to acrolein concentrations ranging from 0 to 1200 μM. After incubating cells with acrolein in cell culture media for 6 h at 37 °C, we observed a decrease in viability starting at 100 μM acrolein and calculated an EC_50_ of 482 μM (Figure 3A). This EC_50_ value is higher than the EC_50_ value of 75 μM found for a glioma cell line exposed to acrolein^43^ and also Caco2 cells for which 30 μM acrolein resulted in ∼40% cell death.^6^ The difference in sensitivity is likely due to differences in cell lines, growth conditions, and/or exposure time. For example, the Caco2 cells were exposed for 24 h, while glioma cells were exposed for 4 h.^6,43^ For further experiments we selected acrolein concentrations ranging between 0 and 100 μM as subcytotoxic concentrations of acrolein to investigate other cell responses.

As a marker for redox homeostasis, we observed a dose-dependent decrease in glutathione levels, with a more than 50% decrease after exposure to 100 μM acrolein (Figure 3B). SW480 cells were exposed to 0 to 100 μM acrolein for 6 h and dl-buthionine-(S,R)-sulfoximine (BSO), a glutathione synthase inhibitor,^44^ was used as a positive control. Glutathione levels were similar in cells exposed to either 500 μM BSO or 75 μM of acrolein. These results support the capacity of acrolein to reduce glutathione levels in SW480 cells, causing redox imbalance and oxidative stress.

### Acrolein Degrades in Cell Growth Media

**Figure 4:**
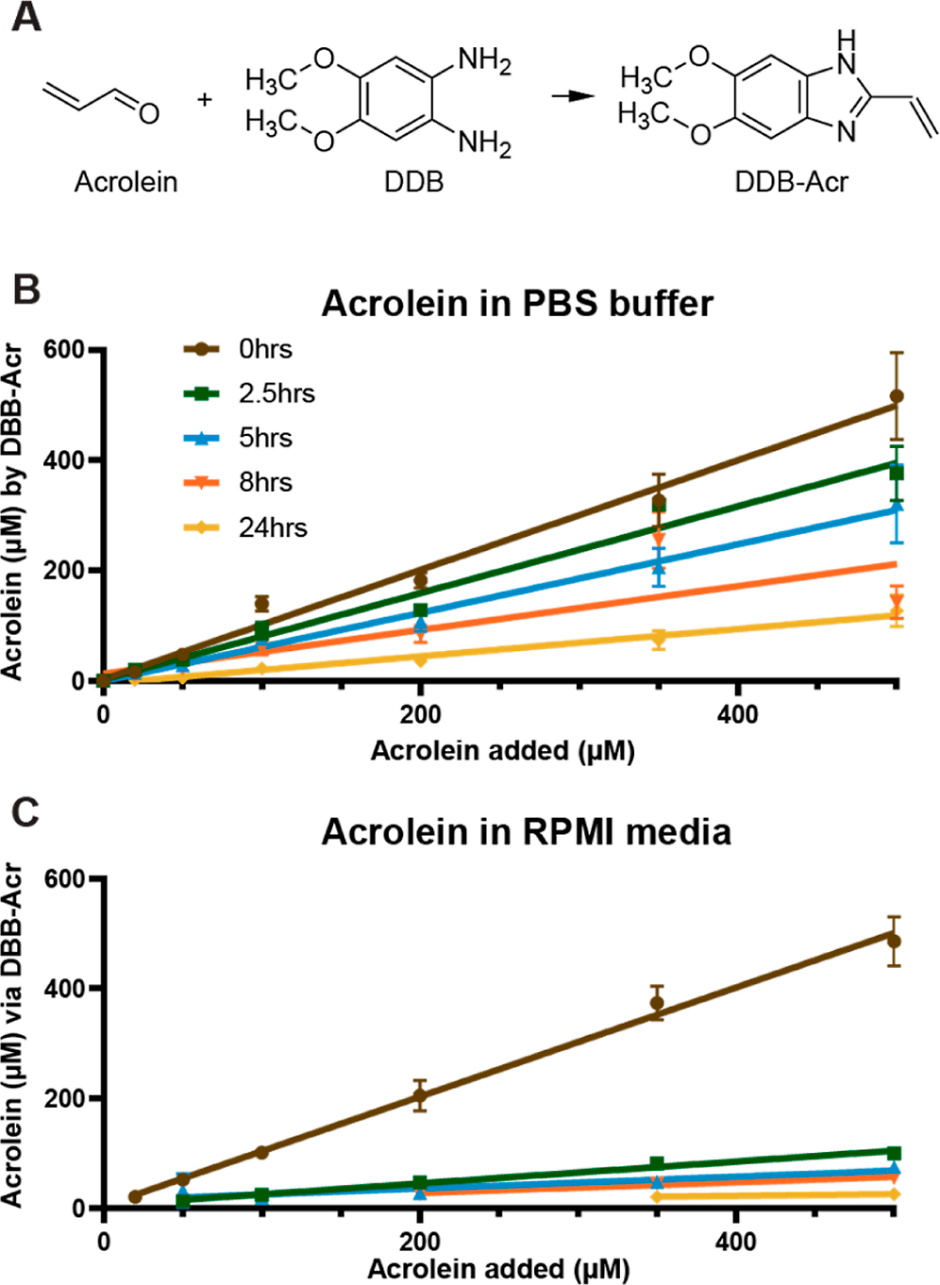
(A) Reaction of acrolein with DBB form DBB-Acr. (B) Acrolein concentration in (B) phosphate buffer and (C) cell culture (RPMI) media without FCS at multiple time points measured by DBB-Acr adduct formation.

To evaluate the available acrolein to form chemical adducts in buffer or media, we measured the conjugate formed from reaction of acrolein with DDB(DDB-Acr) (Figure 4A).^29^ The results (Figure 4B) suggest that acrolein concentrations decrease in sodium phosphate buffer (PBS) with a half-life of 7.2 h. Furthermore, the half-life of acrolein in cell culture media was 2.8 h (Figure 4C). These results are in agreement with another study that concluded the high reactivity and volatility of acrolein contribute to its lack of stability in media.^45^ The accelerated decay in cell culture media compared to buffer suggests it is reacting with cell culture media components. These results indicate a rapid decrease in available acrolein in aqueous solution and that it is quickly depleted in media.

### Viability of Colon Cells with the Enzymatic Acrolein Production System

**Figure 5:**
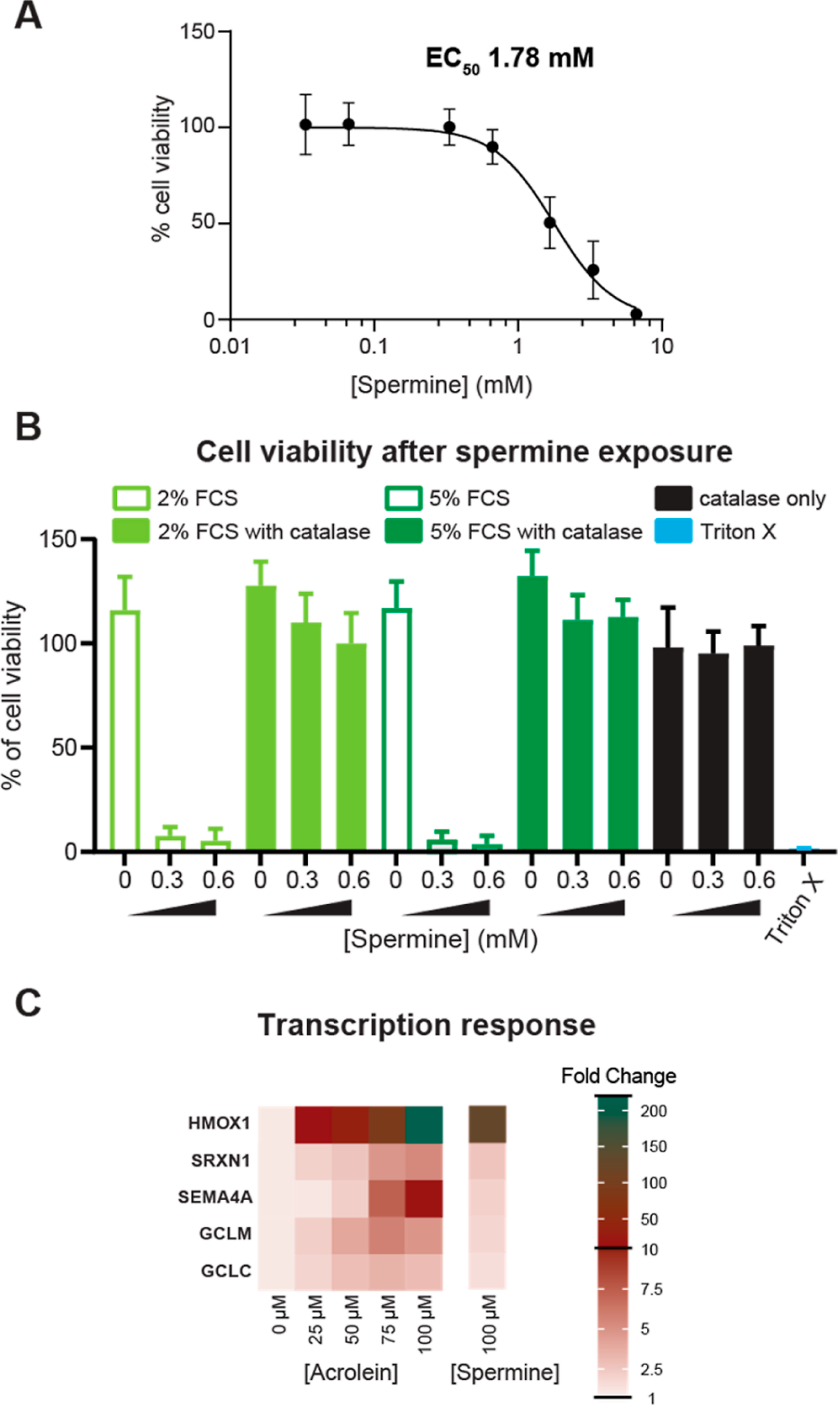
A. Cell viability of SW480 cells exposed to spermine in the enzymatic acrolein production system. Cells were exposed to spermine at 0, 0.3, or 0.6 mM in RPMI media without FCS for 6 h at 37 °C. B. Cell viability of SW480 cells exposed to components of the enzymatic acrolein production system. Cells were exposed to system components in RPMI media with FCS at 2% (light green), 5% (dark green) or 0% FCS (black) for 6 h at 37 °C. In nonfilled bars media was not supplemented with catalase, and in filled bars catalase was added (0.17 mg/mL). C. Transcription levels of oxidative stress related genes in SW480 cells exposed to acrolein, or the enzymatic acrolein production system with spermine supplementation. Average (*n* = 3) transcript levels after exposure to 0, 25, 50, 75, 100 μM acrolein or 100 μM spermine in the enzymatic acrolein production system for 6 h. Each transcript level was normalized to control cells after 0 μM acrolein/spermine and expressed as fold change compared to control.

To verify the biocompatibility of all components of the enzymatic acrolein production system, we performed cell viability experiments, testing combinations of the components. Increasing concentrations of only spermine (6.65 μM – 6.65 mM) in RPMI media without FCS had an EC_50_ value of 1.78 mM (SW480 cells, 6 h exposure, Figure 5A), which is similar to a previous analysis, in which cytotoxicity was observed above 1 mM spermine in a colon carcinoma cell line.^46^ Based on these results, we maintained spermine concentrations below 660 μM for further experiments. Then, to evaluate the combination of acrolein and hydrogen peroxide, we exposed SW480 cells to FCS and spermine with and without catalase (Figure 5B). Cells grown in the presence of FCS and spermine showed a dramatic decrease in cell viability to less than 12% for 0.3 and 0.6 mM spermine conditions, while adding catalase preserved cell viability (Figure 5B). These results suggest that hydrogen peroxide production in this enzyme system has potent cytotoxic effects, which are blocked by adding catalase. Examining the levels of the amine oxidase source, i.e. FCS, we found that with a combination of 2 or 5% FCS with spermine and catalase, cell viability decreased with increasing spermine concentrations. Both 2 and 5% FCS conditions had the same effect on cell viability (Figure 5B), so we chose to use 2% FCS for further experiments. Catalase and spermine without FCS was also tested, confirming they did not reduce cell viability (Figure 5B). These results support the biocompatibility of the extracellular enzyme system and emphasize the importance of removing hydrogen peroxide from the system to observe effects specific to acrolein.

We further compared effects of the enzymatic acrolein production system vs pure acrolein on cell viability. For 600 μM spermine, there was a 25% decrease in cell viability at (Figure 5B, 2% FCS), which was equitoxic with exposure to 250 μM acrolein (Figure 3A). This suggests that for a given spermine concentration, the enzymatic acrolein production system has about ∼40% of the potency of an equivalent concentration of pure acrolein. Given the well-established difficulty in direct measurement of acrolein in cells,^47^ these data are valuable because even without determining the specific acrolein uptake, we could calibrate a molecular indicator for changing acrolein levels and clearly demonstrate the intracellular presence of the chemical. Other variables such as exposure time and media type are likely to influence the response of pure acrolein compared to enzymatically produced acrolein, however, these observations provided a basis for characterizing key molecular responses to the enzymatic acrolein production system.

### Transcriptional Response in Colon Epithelial Cells after Acrolein Exposure

Having established conditions for evaluating effects specific to acrolein and confirming that cells tolerate all components of the enzyme system, we next investigated transcription responses after exposure to acrolein or the enzymatic acrolein production system. Acrolein induces oxidative stress,^27^ thus we selected 5 genes involved in the oxidative stress response (*HMOX*1, *SRXN*1, *SEMA*4A, *GCLM*, *GCLC)*. We compared gene expression from unexposed control cells to acrolein- or the enzymatic acrolein expression system-exposed cells. HMOX1 was the most highly upregulated transcript in response to acrolein exposure with an average of 220-fold increase in cells exposed to 100 μM acrolein compared to control cells (Figure 5C). When cells were exposed to 100 μM spermine in the enzymatic acrolein production system, HMOX1 was also the most upregulated transcript of the analyzed genes, increasing 125-fold (Figure 5C). HMOX1 is a biomarker of oxidative stress^48^ and degrades heme to biliverdin, which cycles between reduced and oxidized forms serving as a potent antioxidant.^49^ Further, SRXN1 increased by 5-fold and 3-fold after exposure to 100 μM acrolein or 100 μM spermine in the enzymatic acrolein production system (Figure 5C). In a previous study SRXN1 also increased after human fibroblasts were exposed to 25 μM acrolein.^50^ SEMA4A increased by 16- and 2-fold compared to control cells in acrolein or enzyme system exposures, respectively (Figure 5C). SEMA4A is a transmembrane protein involved in response to oxidative stress,^51^ but also functions in immune system signaling and angiogenesis.^52^ While acrolein and the enzymatic acrolein production system increased expression of SEMA4A, a much higher response was observed in after exposure to pure acrolein. We also observed a 4-fold and 2-fold increase in GCLM transcription and a 1.5 and 3-fold increase in GCLC transcription after exposure to 100 μM acrolein or 100 μM spermidine in the enzymatic acrolein production system, respectively (Figure 5C). GCLM and GCLC are subunits of the heterodimer protein for glutathione synthesis, and GCLC has previously been shown to increase in HepG2 cells in response to acrolein exposure.^53,54^ This increase in glutathione synthesis genes is expected as glutathione is an antioxidant, and glutathione levels were shown to be reduced during acrolein exposure (Figure 2C). These results indicate that SW480 oxidative stress-related transcriptional responses to acrolein exposure are similar to what has been observed previously in various cell lines, and moreover, that enzymatically produced acrolein from the enzymatic acrolein production system, has similar trends as exposure to pure acrolein.

### DNA Adduct Formation with Acrolein or the Enzymatic Acrolein Production System

**Figure 6:**
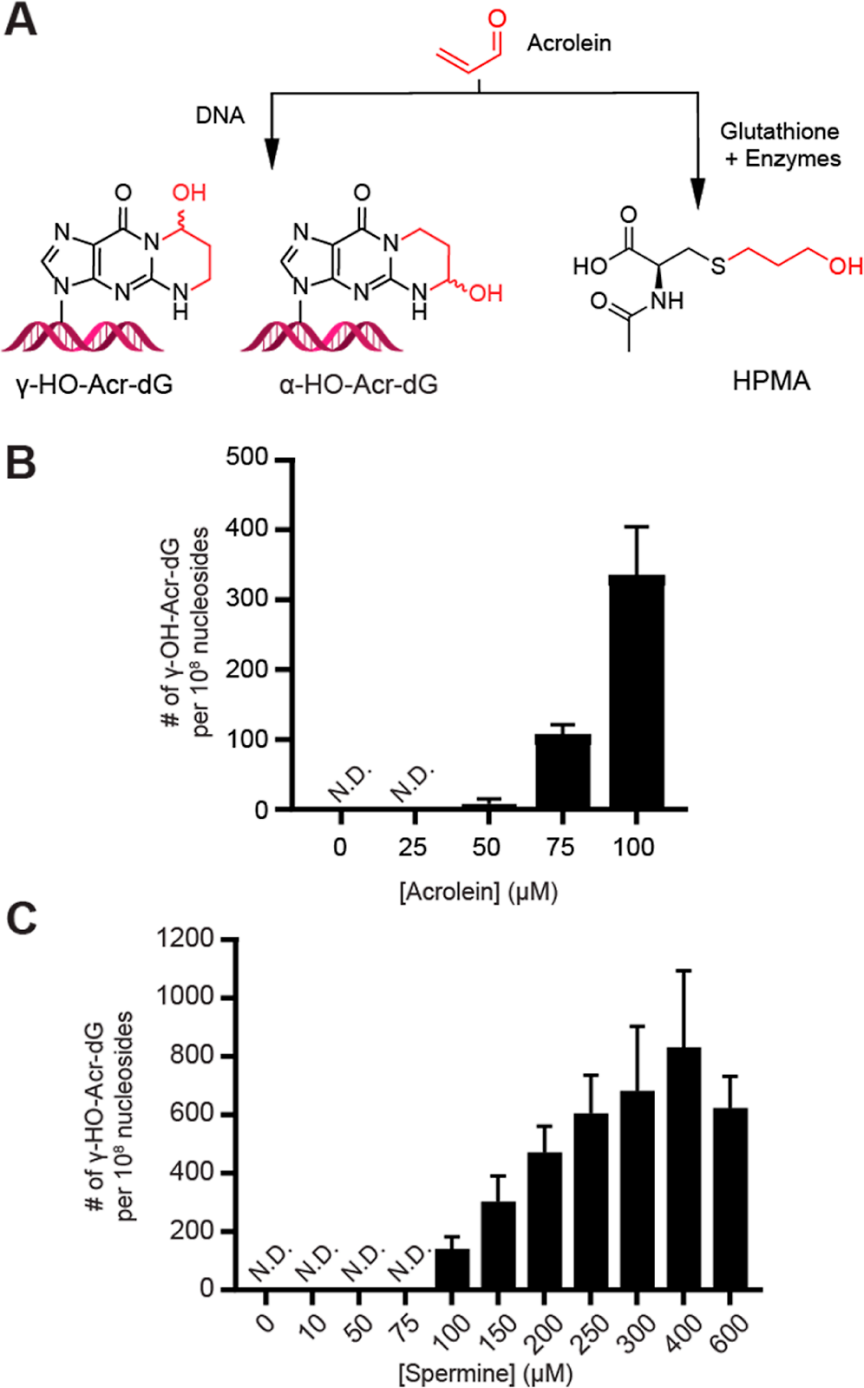
(A) Acrolein reacts with DNA to form two regioisomers of acrolein-dG adducts and acrolein reacts with glutathione to form 3-hydroxypropylmercapturic acid (HPMA). (B) Levels of γ–HO–Acr-dG adducts in SW480 cells after exposure to 0–100 μM acrolein in RPMI media without FCS for 6 h at 37 °C (*n* = 4). DNA was extracted and hydrolyzed, and γ–HO–Acr-dG was quantified by LC–MS/MS. (C) Levels of γ–HO–Acr-dG levels in SW480 cells after exposure to 0–600 μM spermine in the enzymatic acrolein production enzyme system for 6 h at 37 °C (*n* = 4).

Acrolein reacts with deoxyguanosine in DNA, forming regioisomeric HO-Acr-dG adducts (Figure 6A). To further compare cellular responses to the enzymatic acrolein production system vs pure acrolein, we measured the formation of DNA adducts in colon cells. For both exposures we observed γ–HO–Acr-dG formation in a dose-dependent manner, but no α–HO–Acr-dG (Figure 6B and C). The exposure concentration thresholds for quantifying γ–HO–Acr-dG were 50 μM acrolein and 100 μM spermine in the enzymatic acrolein production system, corresponding to 6 and 103 adducts per 10^8^ nucleosides, respectively (Figure 6B and C). Examples of previously reported levels of γ–HO–Acr-dG include DNA from leukocytes of smokers (74 ± 34 adducts per 10^8^ nucleosides) and nonsmokers (98 ± 55 adducts per 10^8^ nucleosides),^10^ as well as human placental DNA (108 ± 26 adducts per 10^8^ nucleosides).^55^ In our experiments, these DNA adduct levels fall between 50 and 75 μM of pure acrolein exposure, and between 75 and 100 μM of spermine addition in the enzymatic acrolein production system (Figure 5B and D). Thus, the enzymatic acrolein production system reaches acrolein levels sufficient to produce biologically relevant levels of acrolein-DNA adducts. Interestingly, the level of acrolein-DNA adducts found in vivo were similar to levels achieved with exposure to 50 μM acrolein and 100 μM spermine in the enzymatic acrolein production system, however acrolein is expected to be present at much lower concentrations in vivo.^56^ Explanations for the higher levels of acrolein necessary to achieve biologically relevant acrolein-DNA adducts are reduced bioavailability of acrolein in cell growth media as presented above, and relatively short-term exposures performed in cell culture compared to accumulation of DNA adducts during the lifetime of cells in the human body. Comparison of DNA adduct formation from acrolein, or the enzymatic acrolein production system was then considered. Exposure of 100 μM of pure acrolein to genomic DNA resulted in an average of 332 γ–HO–Acr-dG per 10^8^ nucleosides (Figure 6B), while 100 μM of spermine in the enzymatic acrolein production system formed 140 γ–HO–Acr-dG per 10^8^ nucleosides (Figure 6C). This suggests that the enzymatic acrolein production system forms ∼40% of the DNA adducts with the equivalent concentration of spermine compared to pure acrolein.

## Conclusions

Herein we developed an extracellular acrolein biosynthesis system comprised of amine oxidase present in FCS, with addition of spermine as substrate, and catalase to remove hydrogen peroxide byproduct, in order to continuously produce acrolein compatible with human cell culture conditions and measure cellular and molecular responses to acrolein. We optimized, validated and calibrated the function of this enzymatic acrolein production system by characterizing changes in cell viability, transcriptional responses, and DNA adduct formation in human cells exposed to pure acrolein and the enzymatic acrolein production system. We established that spermine and FCS concentrations in growth media can be modified to reach desired acrolein exposure levels that achieve biologically relevant DNA adduct levels and modify cell transcription consistent with direct acrolein exposure. This method is expected to enable future experiments to address the host effects of microbially produced acrolein in the intestinal tract and other physiologically relevant sources.

## Supporting Information Available

Additional materials and methods procedures including detailed conditions used in these experiments (DOC) (PDF)
