# Supplementary material for: Enzymatic Acrolein Production System and Its Impact on Human Cells
Source: Chem Res Toxicol. 2024 Jul 9;37(8):1374–81. doi: 10.1021/acs.chemrestox.4c00119 (PMC11337209; doi:10.1021/acs.chemrestox.4c00119)
Supplement: Supplementary file 1 — tx4c00119_si_001.pdf [file tx4c00119_si_001.pdf]

Supporting Information

Enzymatic acrolein production system and its impact on human cells

Katherine A. Hurley, Jacob Folz, Jasmin Zgraggen, Tania Cruz,
Sabine Diedrich, Shana J. Sturla*

[1]Laboratory of Toxicology, Department of Health Sciences and Technology, ETH Zurich, 8092 Zurich, Switzerland

*Correspondence to:
Shana J. Sturla
E-mail: sturlas@ethz.ch
Phone: +41 44 632 91 75
Fax: +41 44 632 11 23

Materials and Chemicals

Material ordered from **Abcam**: WST-1 Cell Proliferation Reagent (Ab155902). Kits and reagents that were ordered from **Sigma**: Acrolein (89116 / Lot#BCBZ3783), putrescine dihydrochloride (P7505-25G / Lot #BCBX2159). Acrolein should always be used in a fume hood to avoid inhalation, o-dianisidine dihydrochloride (F5803-50MG/ Lot #SLBT8735), peroxidase from horseradish (P8250-5KU / Lot #SLBX3736), spermine (S4264 / Lot #BCBW5415), diamine oxidase (D7876 / Lot #011M7015), 4,5-dimethoxy-1,2-phenylenediamine hydrochloride (DDB) (36271/Lot #BCBP3729), catalase from bovine liver (C1345/Lot#SLBZ7596), hydrogen peroxide (216763 / Lot #SZBE0710V), deoxyribonucleic acid sodium salt from calf thymus (D1501-1G/ lot #SLBW1517), Roche DNA Isolation Kit for Cells and Tissue (11814770001/ Lot# 28387500), L-glutathione, reduced (G4251/ Lot# SLCB5110), 2-propanol ≥99.8% (33539-2.5L-R/lot# STBJ7863), DL-buthione-(S,R)-sulfoximine (B2640), ethyl Alcohol molecular biology grade ≥99.8% (459844). Kit and material from **Qiagen:** mini-RNeasy kit (74104), 0.1 mL strip tubes and caps (250) (981103). Kit from **ThermoScientific:** High-capacity cDNA reverse transcription kit (4368814). Materials orders from **VWR**: Centrifugal filters Modified PES 10k (516-0230/lot# FG2056), FilterBio PES sterile syringe filter (FBS13PES022S/lot# 20190925001). Cell culture reagents ordered from **cytiva:** HyClone FetalClone II Serum (U.S.) Heat-inactivated (SH30066.03HI) **Life technologies**: RPMI medium 1640 GLutMAX Gibco (61870-010/lot# 2268106), 0.25% trypsin EDTA Gibco (25200-072/lot# 2212503), fetal bovine serum Gibco (10270-106/lot# 42Q0008K), penicillin streptomycin Gibco (15140-122), Dulbecco's phosphate buffered saline 1x Gibco (14190-094/lot# 2241140). SPE columns were ordered from **Waters**: Oasis HLB 1 cc 30 mg extraction cartridges (186000252/lot# 173B30094B). Material ordered from **Bioswisstec**: Cell scraper PP, 20 mm blade (800030), serological pipette (515210), 15 ml conical tube (50015). Kits from **Promega**: CellTiter-Glo Luminescent Cell Viability Assay (G7571 / Lot #0000378148), GSH/GSSG-Glo Assay (V6611). Materials from **Phenomenex:** HPLC column: Luna 5 µm C18(2) 100 Å 250 x 4.6 mm Analytical (00G-4252-E0); LCMS Column: Luna 5 µm C18(2) 100 Å, 250 mm length x 0.5 mm i.d. Capillary (00G-4252-AF). Materials from **Solis BioDyne**: 5x HOT FIREPol EvaGreen qPCR Mix Plus (ROX) (08-24-00001).

Cell growth conditions

Human colorectal adenocarcinoma cells (SW480) were cultured in complete RPMI medium 1640 1x + GLutMAX supplemented with 10% fetal bovine serum (FBS) and with 1% penicillin streptomycin (pen/strep) (RPMI+FBS) as a monolayer in 10 cm dishes or 96-well plates and incubated at 37 °C with 5% humidity for 72 h. During exposure experiments, cells were cultured in RPMI Medium 1640 (1x) + GLutMAX supplemented with FBS and without 1% pen/strep (RPMI-FBS) in a humified 5% CO_2 atmosphere.

After exposure the cells were harvested by scraping them off the bottom of the 10 cm dishes using a 20 mm rubber blade with buffer or lysis solution.

Cell viability after exposure to acrolein or the enzymatic acrolein production system. SW480 cells were seeded at a density of 10,000 cells per well in a 96-well plate in RPMI media with FBS and incubated until 80-90% confluency was reached (72hrs). A 200 mM stock solution of acrolein in Milli-Q water was made in a fume hood and then filtered with a 0.22 micron sterile filter to ensure sterility. The 200 mM stock solution was further diluted to make working stock solutions in RPMI without FBS to give the final exposure concentrations of 2000, 500, 250, 100, 50, 25, 10, 5, 2, and 1 µM. A 100 mM stock solution of spermine was prepared in RPMI without FBS and then filtered with a 0.22 micron sterile filter. The 100 mM stock solution was further diluted to make working stock solutions in RPMI without FBS to give the final exposure concentrations of 6.65, 3.32, 1.66, 0.66, 0.33, 0.06, and 0.03 mM. Cells were washed with 100 µL of 1 x PBS and acrolein or spermine solutions were added to the 96 well plate and the plate was incubated for a total of 6 h at 37 °C. A volume of 100 µL of 500 µM hydrogen peroxide in RPMI without FBS was used as a positive control. After 5hrs of incubation at 37 °C, 10 µL of WST-1 cell proliferation reagent was added to each well and mixed by shaking the plate on a flat surface for 30 s. After an additional 1 h of incubation at 37 °C, the absorbance was measured on a Tecan Infinite M200 PRO plate reader at 440 nm. Percent cell viability was normalized to ”media only” condition as 100% and calculated using the equation below. The cell viability curve was fit using Prism GraphPad software.

$$Cell\,Viability\,(\%) = \frac{average\ of\ (X\ -\ average\ of\ background)}{average\ of\ negative\ control} \times 100$$

Exposure of SW480 cells to acrolein for DNA extraction. SW480 cells were seeded in 15 cm plates with 8-10x10^6 cells per dish in RPMI media with FBS and incubated until a density of between 40-50x10^6 cells per dish was reached. A 200 mM stock solution of commercially available acrolein in Milli-Q water was prepared in a fume hood and then filtered with a 0.22 micron sterile filter to ensure sterility. The 200 mM stock solution was further diluted to a 2 mM working stock in RPMI without FBS media. The 2 mM working stock solution was diluted to the final exposure concentrations of 0, 25, 50, 75 or 100 µM in 20 mL in 50 mL conical tubes. The old RPMI with FBS media was removed from the culture dishes, acrolein solutions added, and plates incubated at 37 °C for 6 h. After incubation, cells were washed once with 20 mL of PBS while still attached to the dish to remove the acrolein. A volume of 600 µL of 10 mM glutathione (GSH) in milli-Q water was added to 11.4 mL of cell lysis buffer from Roche DNA Isolation Kit to a final concentration of 0.5mM GSH to scavenge any unreacted acrolein. The cell lysis buffer supplemented with GSH was added to each plate. The cells were then scraped and

transferred to a 15 mL conical tube using a serological pipette. Cell lysates were sonicated for 5 min to ensure cells were fully lysed and then samples were stored at -20°C until processing with Roche DNA Isolation kit.

Exposure of SW480 cells to acrolein for RNA extraction. SW480 cells were seeded in 10 cm plates with $3x10^6$ cells per dish in RPMI media with FBS and incubated until a density of 80-90 % confluency was reached. For the acrolein exposure, a 200 mM stock solution of commercially available acrolein in Milli-Q water was prepared in a fume hood and then filtered with a 0.22 micron sterile filter to ensure sterility. The 200 mM stock solution was further diluted to a 2 mM working stock in RPMI. The 2 mM working stock solution was diluted to the final exposure concentrations of 0, 25, 50, 75 or 100 µM with RPMI media without FBS to 10 mL. The old RPMI with FBS media was removed from the culture dishes and the acrolein solutions were added to the dish. The dishes were incubated at 37 °C for 6 h. After exposure, the media was aspirated, and cells were washed once with 10 mL of PBS while still attached to the dish. A volume of 10 mL of PBS was added to the dish and cells were harvested by using a cell scraper. The PBS containing the cells was transferred to a conical tube and the dish was washed with additional 2 mL of PBS. The cells were spun down at room temperature for 4 min at 4000 rpm. The supernatant was aspirated, and the cell pellet were kept at -80 °C until RNA extraction.

Glutathione measurement after SW480 cell exposure to acrolein or BSO. SW480 cells were seeded in 96 well plates with $0.01x10^6$ cells per well and incubated until a density of 80-90 % cells per well was reached. A 10 mM stock solution of DL-buthione-(S/R)-sulfoximine was prepared in RPMI without FBS and then filtered with a 0.22 micron sterile filter to ensure sterility. The stock solution was diluted to a final concentration of 500 µM with RPMI without FBS. A 200 mM stock solution of acrolein was prepared in Milli-Q water and then filtered with a 0.22 micron sterile filter. The 200 mM stock solution was further diluted to a 2 mM working stock in RPMI without FBS, which was used to make the final exposure concentrations of 0, 25, 50, 75 or 100 µM acrolein in RPMI without FBS. All solutions were prepared in 1.5 ml sterile Eppendorf tubes in RPMI without FBS to a final volume of 100 µl per well. The negative control was RPMI without FBS. The old media was removed from the 96 well plates, and then the solutions of BSO and acrolein were added and the 96 well plates were incubated at 37 °C for 6 h. After incubation, the GSH and GSSG levels were measured following the protocol provided in the GSH/GSSG-Glo Assay kit (Promega).

DNA extraction. Frozen cell lysate samples were processed using the Roche DNA isolation kit for cells and tissues with some adjustments to the manufacturer’s protocol. Tubes with cell lysates were thawed in a 37 °C water bath for no more than 5 min and

then allowed to fully thaw at room temperature (RT). A volume of 8 µL of proteinase K solutions provided by the kit was added, and then tubes were inverted and incubated in a water bath at 65 °C for 2 h. Samples were removed from water bath and allowed to cool to 25 °C (~5 min). A volume of 400 µL of RNase solution provided by the kit was added. Tubes were then inverted several times and then incubated at 37 °C for 1 h. Protein precipitation solution (PPS) was supplemented with 10 mM GSH in milli-Q water to a final concentration of 0.5 mM GSH (PPS+GSH). A volume of 5 mL of PPS+GSH was added to high centrifugation Nalgene tubes chilled on ice. Cell lysates were then added to the Nalgene tubes containing PPS+GSH, vortex-mixed for 15-30 min at 37 °C, and incubated on ice for 5 min. Tubes were centrifuged at 26,900 *g* at 20 °C for 20 min. The supernatant was transferred to a clean 50 mL conical tube and 17.408 mL (0.7 volumes) of isopropanol was slowly added to each sample by allowing the solvent to slowly flow down the inside of the tube. Tubes were then slowly inverted to mix the upper and lower phases until DNA precipitates formed. Tubes containing extracted DNA were centrifuged at 1,370 g-force for 10 min and supernatant was discarded. The DNA pellet was washed by adding 10 mL of ice cold 70% ethanol, and then tapping the tube to dislodge the pellet to float in the ethanol. The samples were centrifuged for 5 min at 1,370 g-force and supernatant was discarded. The excess ethanol was allowed to evaporate, but the DNA pellet was not allowed to dry completely. The DNA pellet was resuspended in DNA hydrolysis buffer (10 mM sodium succinate, 5 mM $CaCl_2$, 0.5mM GSH at pH 7.0). DNA amount was quantified with Quantus Fluorometer and OneDNA fluorescent dye. The dissolved DNA was stored at -20 °C.

DNA hydrolysis. DNA extracted from exposed cells was defrosted on ice and enzymatically hydrolyzed to a mixture of single nucleosides. A mass of 60 µg of extracted DNA was added to a reaction mixture of 0.5 mM of GSH with 10 nM of isotopically labeled internal standards ([$^{15}N_5$]-α-OH-Acr-dG and [$^{15}N_5$]-γ-OH-Acr-dG, in DNA hydrolysis buffer (10 mM sodium succinate, 5 mM $CaCl_2$ at pH 7)). The mixtures were heated to 100 °C for 30 min with shaking at 500 rpm using a thermal shaker (Eppendorf). The mixtures were removed from the heat and allowed to cool for 10 min. Seventy-five units of micrococcal nuclease (stock concentration of 15 U/µL in storage buffer 10 mM Tris-HCL, 50 mM NaCl, 1 mM EDTA, 50% glycerol stored at -80 °C) and 0.45 units of phosphodiesterase II (stock concentration of 0.05 U/µL in DNA hydrolysis buffer stored at -20 °C) were added to the reaction mixtures. The reactions were mixed by pipetting and incubated for 6 h at 37 °C with shaking at 500 rpm. After 6 h, 150 units of alkaline phosphatase (used from commercial bottle and stored at 4 °C) was added to the reaction mixtures. The reactions were mixed with pipetting and incubated overnight at 37 °C with shaking at 500 rpm. Reaction tubes were cooled to room temperature and the mixture was transferred to a 10 kDa centrifuge filter. The solutions were centrifuged at 14,000 rpm for 15 min to remove proteins from the reaction mixture. A volume of 25

µl of the filtered mixture was removed and stored at -20 ᵒC for guanosine quantification by HPLC. The remaining mixture was purified using solid-phase extraction (SPE) cartridges (Oasis HLB 1 cc Vac Cartridges, 30 mg sorbent, 30 uM particle size (Waters)). SPE cartridges were activated with 1 mL of LCMS grade methanol, 1 mL of 50:50 LCMS grade methanol with 0.1 mM GSH in LCMS grade H_2O, and 1 mL of 0.1 mM GSH in LCMS grade H_2O. The reaction mixtures were added to the activated cartridge and washed with 1 mL of 0.1 mM GSH in LCMS grade H_2O. The nucleosides were eluted from SPE cartridge with 1 mL of methanol and collected in Lobind DNA tubes. A volume of 10 µL of 10 mM of GSH in LCMS grade H_2O (100 nmol of GSH) was added to the collected methanol eluents to scavenge any unreacted acrolein. The eluents were evaporated to dryness using a speed vacuum concentrator. The samples were stored at -20 ᵒC until LCMS analysis.

Quantification of deoxyguanosine by HPLC for normalization of OH-Acr-dG. A mixture of standard deoxynucleosides (dNTPs) was used as a quality control for nucleoside separation. Ten mM stocks of each 2'Deoxyguanosine monohydrate (dG), 2'-Deoxyadenosine monohydrate (dA), 2'-Deoxycytidine (dC), and 2'-deoxythyminine (dT) were prepared in water and 1 µL of each was added to 996 µL of Milli-Q water. The dG calibration standards for quantifying the unknown dG in the hydrolyzed samples were prepared at 0.1, 1, 5, 10, 25, 50 µM in water. The 25 µL sample aliquots collected after enzymatic DNA hydrolysis were diluted with 50 µL of Milli-Q water for analysis. HPLC solvents were (A) Milli-Q water and (B) Methanol. The HPLC was equipped with a Luna 5 µm C18(2) 100 Å Phenomenex column (250 x 4.6 mm) absorbance at 254nm was monitored with an injection volume of 20 µL. The calibration standards were injected before and after each set of samples. For each standard and sample, the peak area of the dG was integrated. A calibration curve was constructed using the peak area of the standards compared to the known dG concentrations. A linear regression equation was then applied to calculate the total number of dG nucleosides in each sample.

Quantification of HO-Acr-dG by LCMS. Dried and hydrolyzed DNA samples were dissolved in 20 µL of LCMS grade water and incubated on ice for 10 min. Samples were centrifuged at 4 °C for 15 min at 14000 rpm. A volume of 15 µL of supernatant was removed and transferred to LCMS vials for analysis. Calf thymus DNA (CT DNA) was hydrolyzed as matrix for LCMS calibrants. The dried CT DNA samples were dissolved in 20 µL of calibrant solutions in LCMS grade water giving final concentrations of 0, 4, 8, 16,32, and 128 nM α-OH-Acr-dG and γ-OH-Acr-dG with 10 nM of internal standards [$^{15}N_5$]-α-OH-Acr-dG and [$^{15}N_5$]-γ-OH-Acr-dG. Analytes α-OH-Acr-dG and γ-OH-Acr-dG in the samples were analyzed using an Acquity ultra performance liquid chromatography (UPLC) instrument (Waters) and TSQ Vantage (Thermofisher) triple

quadrupole mass spectrometer. A Luna C18(2) (0.5mmi.d. x 250 mm length; 5 µm particle size, pore diameter 100 Å) column (Phenomenex). The autosampler was cooled to 4 °C, column oven was set to 40 °C, flow rate of 10 µL/min, and 2 µL injection volume with with full loop injections. α-OH-Acr-dG and γ-OH-Acr-dG both presented the transition of 324 (m/z) to 208 (m/z) due to the loss of the sugar moiety. The α-OH-Acr-dG stereoisomers were observed as two separate peaks. γ-OH-Acr-dG stereoisomers were observed as one peak. Further fragmentation of the γ-OH-Acr-dG resulted in the loss of both the sugar and the γ-hydroxyl group resulting in transition of 324 (m/z) to 164 (m/z). For the [$^{15}N_5$]-labeled α-OH-Acr-dG and γ-OH-Acr-dG internal standards, the same peak retention times and fragmentation patterns were observed compared to unlabeled standards, however the mass transitions were 5 m/z higher. The mass transition of 329 (m/z) fragmented to 213 (m/z) due to the loss of the sugar and the [$^{15}N_5$]-γ-OH-Acr-dG further fragmented to 169 (m/z) from 329 (m/z). Peak integration was performed using Xcalibur software (ThermoFisher Scientific) and a peak area ratio was calculated by dividing the peak area of the α-OH-Acr-dG or γ-OH-Acr-dG by the peak area of the respective internal standard. Linear regression curves were used to calculate the concentrations of α-OH-Acr-dG and γ-OH-Acr-dG in samples. The number of α-OH-Acr-dG and γ-OH-Acr-dG was calculated and normalized to measured dG values to give number of α-OH-Acr-dG or γ-OH-Acr-dG per 10^8 nucleosides.

RNA extraction and reverse transcription. Frozen cell pellets from 10 cm dishes were thawed on ice and resuspended in 1 mL of PBS. Volumes of 600 µL of the resuspended cells were aliquoted into new tubes and then the RNA extraction was performed. Total RNA was isolated using the mini-RNeasy RNA isolation kits (Qiagen) according to the manufacturer's instructions. During the cell lysis step, the SW480 pellet was broken up with additional vortexing for 15 seconds and pipetting using a 1 mL pipet until the tip did not clog. The RNA concentration was assessed by UV absorption on a Nanodrop spectrophotometer (Thermofisher). Masses of 1 µg or 2 µg of RNA were retrotranscribed using the High-Capacity cDNA Reverse Transcription Kit (ThermoFisher Scientific) according to the manufacturer's instructions. The retrotranscription program was as follows: 25 °C for 10 min, 37 °C for 120 min, 85 °C for 5 min. The complementary DNA (cDNA) aliquots were then diluted to obtain working solutions with a cDNA concentration of 2 ng/µl (1:25 for 1 µg and 1:50 for 2 µg cDNA), and stored at -20 °C until use.

Quantitative polymerase chain reaction. Primers for qPCR were designed with the Universal Probe Library (Roche) online software, using an intron-spanning assay, and optimizing the melting temperature to 60 °C. Primers were screened for self-complementarity using the Oligo Calculator and localization on target RNAs with NIH PrimerBlast. The primers were obtained from Eurogentec and their sequence are

reported in Table S1. The qPCR reaction mixtures contained ~6 ng cDNA, 0.4 μM of forward and reverse primers, and the HOT FIREPol EvaGreen 5x mastermix (Solis Biodyne). The thermal cycling protocol for the qPCR analyses included the following steps: initial melting step at 95 °C for 5 min, 50 cycles of 1) melting at 95 °C for 30 s, 2) annealing and extension at 60 °C for 30 sec. At the end of each cycle the fluorescence of HOT FIREPol EvaGreen was measured. After the 50 cycles, a hold step at 72 °C for 5 min was added to ensure the proper completion of the qPCR program. To determine the melting temperature of the amplicons and analyse primer specificity, a melting curve was implemented with cDNA samples. The melting curve was obtained by increasing the temperature one degree per min starting at 60 °C and going to 99 °C. The fluorescence emission intensity was measured at the end of each min and remaining qPCR products were monitored. If two peaks were observed, new primer sets were designed. For the qPCR data analysis, a cycle threshold (Ct) value of 0.2 for the fluorescence signal was set and Ct values were obtained. The delta Ct (dCt) values were calculated for each gene by normalizing to the Ct values of the housekeeping gene GAPDH within each experimental condition. The delta delta Ct (ddCt) values were obtained by normalizing the calculated dCt values of each gene to the experimental control conditions. The fold change (FC) values were then calculated by the following equation: $FC = 2^{-ddCt}$. R programming language was used to generate heatmaps of gene transcription variation.

Table S1. primers used in this study

Gene Name	Product Size	Primers
HMOX1	83	Forward: TGCCACACAGCGACAGAG
		Reverse: TTCAGGGCCTCTGACAAATC
SRXN1	71	Forward: GGGTCTAGGGGAAGAGGTGT
		Reverse: CACTCCTACTACAGTGGGTCCAG
SEMA4A	137	Forward: TCCTACCTGTTGCCCATCTC
		Reverse: CTGCCCAGGAAGTTGTTCAT
GCLC	132	Forward: TGCCTATGTGGTGTTTGTGG
		Reverse: ATTCCCTGCAAGACAGCATC
GCLM	98	Forward: GACAAAACACAGTTGGAACAGC
		Reverse: AAATCTGGTGGCATCACACA
GAPDH	231	Forward: ACGGATTTGGTCGTATTGGG
		Reverse: TGATTTTGGAGGGATCTCG

Measurement of cell viability after exposure to the enzymatic acrolein production system. SW480 cells were seeded in 96 well plates with 0.01×10^6 cells per well and incubated until a confluency of 80-90 % was reached. The 10 mM stock solution of the spermine was dissolved in RPMI without FBS media and 1 mg/mL stock solution of catalase was dissolved in 50mM potassium phosphate buffer. Both solutions were

filtered with 0.22 μm filter to ensure sterility for every experiment. The Hyclone FCS was defrosted and warmed to 37 ^oC. A final concentration of 100 μM of acrolein and 500 μM of H_2O_2 were used as positive controls and RPMI without FBS was a negative control for data normalization. The old media was aspirated and the appropriate volume of new RPMI without FBS media was added to all wells depending on the condition of each well. For the conditions with spermine, 3.25 μL or 6.5 μL of the 10 mM stock solution of spermine was added to the wells to give final concentrations of 330 μM or 660 μM. For the conditions with catalase, 16 μL of 1 mg/mL of catalase stock solution was added to the wells to give a final concentration of 0.16 mg/mL. Finally, the enzyme reaction was started by adding 2 μL of Hyclone FCS to give 2% (v/v) Hyclone FCS as the last component. The plates were gently mixed by pipetting and were incubated at 37 °C for 6 h. All conditions tested for the coupled enzyme system are as follows: 1) Hyclone FCS only, 2) Hyclone FCS and spermine 3) Hyclone FCS, spermine, and catalase, 4) catalase only, and 5) catalase and spermine. After exposure time of 6 h, the medium was removed and 20 μL of fresh RPMI without FBS was added. Cell viability was determined using the CellTiter-Glo Luminescent Cell Viability Assay (Promega). CellTiter-Glo reagent were equilibrated to room temperature and 20 μL was added to each well. Solutions were then mixed by shaking for 2 minutes, and further incubated for 10 min at room temperature. After the incubation, 30 μL of the solution in the wells was transferred to a white half-area 96 well plate and luminescence signal was recorded with a Tecan Plate Reader Infinite M200 PRO. Cell viability experiments were performed in three technical and three biological replicates with cells at different passage numbers. Percent cell viability was normalized to “media only” condition as 100% and calculated using the equation below with X being the sample luminescence value.

$$Cell\,Viability\,(\%) = \frac{average\ of\ (X\ -\ average\ of\ background)}{average\ of\ negative\ control} \times 100$$

Enzyme kinetics in the enzymatic acrolein production system in 96-well plates. The enzyme kinetics of Hyclone FCS with spermine was assessed by adapting the diamine oxidase activity assay kit from Sigma-Aldrich using peroxidase and o-dianisidine as a colorimetric readout in 96-well plates.[1] A 40 mM stock solution of spermine was prepared in 100 mM sodium phosphate buffer and put on ice. A 10 mg/mL stock solution of peroxidase was dissolved in cold 100 mM sodium phosphate buffer and put on ice. A 5 mg/mL stock solution of o-dianisidine was dissolved in MilliQ H_2Oin an tube wrapped in foil, and left at room temperature. Hyclone FCS was defrosted and diluted 2- and 5-fold with 100 mM sodium phosphate buffer and left at room temperature. Test concentrations of 10%, 5%, and 2% (v/v) of Hyclone FCS were tested. For conditions with spermine, 20 μL of buffer was added to all the wells of a 96 well plate, except the top row. In the top row, 40 μL of 40 mM of spermine was

aliquoted, then 20 µL was transferred from the top row to the next row containing 20 µL of buffer completing a 2-fold dilution. This was repeated down the rows of the plate to give a 2-fold dilution series of spermine. A volume of 230 µL or 250 µL of 100 mM sodium phosphate buffer was added to wells containing spermine and wells not containing spermine, respectively. A volume of 10 µL of 10 mg/mL of peroxidase (final concentration: 0.33 mg/mL) was aliquoted into each well and mixed by pipetting. The empty wells were filled with 300 µL of 100 mM sodium phosphate buffer to reduce evaporation. In a second 96 well plate, 40 µL of each Hyclone FCS dilution were pipetted into the corresponding well of the conditions in the first 96 well plate. This Hyclone plate was sealed with a plastic film and set aside. In a dark room, 10 µL of 5 mg/mL of o-dianisidine (final concentration: 0.17 mg/mL) was added to each well and mixed by pipetting. The 96 well plate was covered with a lid and incubated for 15 min at 37 °C. At the same time, the Hyclone plate was also incubated for 15 min at 37 °C. After the incubation, using a multichannel pipet, 30 µL of Hyclone FCS was transferred from each row of the Hyclone plate to the first 96 well plate and mixed by pipetting up and down twice with care to avoid bubbles to give 300 µL final reaction volume. The 96 well plate was sealed with a plastic film before being placed in the plate reader. The absorbance was measured at 440 nm every 10 min over 20 h at 37 °C with shaking prior to each measurement using a Tecan Plate Reader (Infinite M200 PRO). For kinetic calculations the data was fit to a non-linear regression curve using GraphPad Prism using Michaelis-Menten function and automatically reported Vmax for each FCS concentration.

Exposure of SW480 cells to the enzymatic acrolein production system. SW480 cells were seeded in 10 cm plates with $3x10^6$ cells per dish in RPMI media with FBS and incubated until a density of 80-90 % confluency ($10\text{-}20x10^6$ cells) was reached (~ 72 h). A 10 mM stock solution of spermine was made in RPMI without FBS media and 1 mg/mL stock solution of catalase was made in 50 mM potassium phosphate buffer. Both stock solutions were filtered with 0.22 µm to ensure sterility for every experiment. The Hyclone FCS was defrosted and warmed to 37 ^{o}C. A final concentration of 100 µM of acrolein was used as positive control and RPMI without FBS was used as a negative control. The old media was aspirated, cells were washed with 1 x PBS, and the appropriate volume of new RPMI without FBS was added to all dishes. For the conditions with spermine, 50, 100, 300, and 600 µL of the 10 mM stock solution of spermine was added dropwise to the dish to give final concentrations of 50, 100, 300, and 600 µM. For the conditions with catalase, 1665 µL of 1 mg/mL of catalase stock solution was added dropwise to the dishes to give a final concentration of 0.16 mg/mL. Finally, the enzyme reaction was started by adding 200 µL of Hyclone FCS to give 2% (v/v) Hyclone FCS as the last component. The final volume for all dishes was 10 mL. The dishes were mixed by moving them in a figure 8 pattern 5 times in both directions.

The dishes were incubated at 37 °C for 6 h. After exposure time of 6 h, the media was aspirated, and cells were washed with 10 mL of PBS. A volume of 10 mL of PBS was added to each dish, cells were scraped off the bottom of the plate, and transferred to 15ml conical tubes using a serological pipet. Another 2 mL of PBS was added to the plates to transfer remaining cells to the same tube. The 15 mL tubes were centrifuged for 4 min at 400 rcf and the supernatant was aspirated. Cell pellets were stored in -80 °C until DNA or RNA extraction.

DDB-Acr derivatization with acrolein in buffer and media. Acrolein stock solutions in water were diluted 1:10 in 100 mM sodium phosphate buffer or RPMI media to a final concentration of 0, 20, 50, 100, 200, 350, and 500 µM. The acrolein solutions were incubated at 37 °C for 24 h and aliquots were taken at 0, 2.5, 5, 8, 24 h. Volumes of 30 µL of acrolein solutions were added to 370 µL of methanol to denature proteins in the sample mixture. After quick vortex-mixing, the mixture was centrifuged at 4 °C for 10 min at 2000 *g*. The supernatant was diluted 1:4 with LCMS grade water and then used for the derivatization with DDB. A volume of 100 µL of the diluted supernatant was added to 100 µL of 1 mM DDB in LCMS grade water. The DDB solution was prepared fresh for every experiment. The mixture was vortex-mixed and heated at 80 °C for 10 min following by centrifugation at 4 °C for 15 min. Volumes of 100 µL of the samples were added to LCMS vials and frozen at -20 °C until LCMS analysis. The experiment was performed in three biological replicates. Peak area was integrated using Xcalibur (version 4.0, Thermo Fisher) and plotted with GraphPad Prism.

LCMS analysis and quantification of acrolein-DDB conjugate. The analysis of the acrolein-DDB conjugate was performed by using a nanoACOUITY™ Ultra-Performance Liquid Chromatography (UPLC) system (Waters Corporation, Milford, MA, USA) equipped with a 5 µL injection loop and coupled to a orbitrap LTQ velos mass spectrometer (Thermo Fisher, San Jose, CA, USA). Separation was performed by using a capillary LC column Synergi™ 4 µm Polar-PR 80 Å (150 mm length x 0.5 mm inner diameter). Mobile phase A was 0.1% formic acid in LCMS grade water and mobile phase B was 0.1% formic acid in acetonitrile. Flow rate of mobile phases was 8 µL/min and the gradient went from 0%B from 0 to 1 minute to 44% B at 18 minutes, 90% B at 19 min, held at 90%B from 19 to 23 min, 0%B by 24 min, and held at 0% B from 24 to 27 minutes. Data was acquired as full scans between 50 to 500 *m/z*. Instrument control and data acquisition were performed using Xcalibur (version 4.0, Thermo Fisher). Samples were injected twice as technical duplicates. The peaks at 13.7 min with m/z of 205 were integrated using a processing method that detects peaks at a S/N of 10 (LOQ). A linear model between acrolein added and DBB-Acr adduct peak area were generated and used to calculate concentration of acrolein in the samples. Half-life of

acrolein was calculated using 500 μM addition of acrolein and all sampled timepoints (2.5 h, 5 h, 8 h, 24 h).

References

(1) Claiborne, A., and Fridovich, I. (1979) Chemical and enzymic intermediates in the peroxidation of o-dianisidine by horseradish peroxidase. 1. Spectral properties of the products of dianisidine oxidation. *Biochem. 18*, 2324–2329.
